# Nucleotides upstream of the Kozak sequence strongly influence gene expression in the yeast *S. cerevisiae*

**DOI:** 10.1186/s13036-017-0068-1

**Published:** 2017-08-21

**Authors:** Jing Li, Qiang Liang, Wenjiang Song, Mario Andrea Marchisio

**Affiliations:** School of Life Science and Technology, Harbin Institute of Technology, 2 Yikuang Street, Nan Gang District, Harbin, 150080 People’s Republic of China

**Keywords:** Synthetic biology, *S. cerevisiae*, 5^′^-UTR, Kozak sequence

## Abstract

**Background:**

In the yeast *Saccharomyces cerevisiae*, as in every eukaryotic organism, the mRNA 5^′^-untranslated region (UTR) is important for translation initiation. However, the patterns and mechanisms that determine the efficiency with which ribozomes bind mRNA, the elongation of ribosomes through the 5^′^-UTR, and the formation of a stable translation initiation complex are not clear. Genes that are highly expressed in *S. cerevisiae* seem to prefer a 5^′^-UTR rich in adenine and poor in guanine, particularly in the Kozak sequence, which occupies roughly the first six nucleotides upstream of the START codon.

**Results:**

We measured the fluorescence produced by 58 synthetic versions of the *S. cerevisiae* minimal *CYC1* promoter (pCYC1min), each containing a different 5^′^-UTR. First, we replaced with adenine the last 15 nucleotides of the original pCYC1min 5^′^-UTR—a theoretically optimal configuration for high gene expression. Next, we carried out single and multiple point mutations on it. Protein synthesis was highly affected by both single and multiple point mutations upstream of the Kozak sequence. RNAfold simulations revealed that significant changes in the mRNA secondary structures occur by mutating more than three adenines into guanines between positions −15 and −9. Furthermore, the effect of point mutations turned out to be strongly context-dependent, indicating that adenines placed just upstream of the START codon do not *per se* guarantee an increase in gene expression, as previously suggested.

**Conclusions:**

New synthetic eukaryotic promoters, which differ for their translation initiation rate, can be built by acting on the nucleotides upstream of the Kozak sequence. Translation efficiency could, potentially, be influenced by another portion of the 5^′^-UTR further upstream of the START codon. A deeper understanding of the role of the 5^′^-UTR in gene expression would improve criteria for choosing and using promoters inside yeast synthetic gene circuits.

**Electronic supplementary material:**

The online version of this article (doi:10.1186/s13036-017-0068-1) contains supplementary material, which is available to authorized users.

## Background

Rational design of synthetic gene circuits demands proper characterization and categorization of their basic components. These basic components are DNA sequences associated with precise transcription or translation functions and are known as standard biological parts [1]. Bacterial parts are divided into four main groups: promoters, ribosome binding sites (RBSs), coding sequences (CDSs), and terminators. Promoters and terminators are, respectively, the start and stop signals for DNA transcription into mRNA. The RBS is the entry point for the ribosomes into the mRNA, i.e. the place where translation initiation takes place. Protein synthesis terminates when the ribosomes meet the STOP codon at the end of the CDS.

The main feature of the RBS is the Shine-Dalgarno sequence, located approximately 10 nucleotides upstream of the START codon. Its complementarity to a ribosomal region allows it to be recognized and bound by ribosomes. The RBS has been widely studied and, remarkably, software has been developed to design regulated RBSs with the aim of tuning gene expression [2].

Eukaryotic cells do not have a counterpart to the Shine-Dalgarno sequence and mature mRNA is recognized by ribosomes because of the presence of the 5^′^ cap. Although the mRNA 5^′^ untranslated region (UTR) plays an important role in determining protein expression [3–5], a eukaryotic standard biological part corresponding to it has never been defined and it is regarded simply as the end of the promoter sequence.

A feature specific to eukaryotic mRNA is the Kozak sequence [6], which extends from approximately position −6 to position +6, where +1 is assigned to the adenine of the START codon (throughout the present paper, all positions are given respective to the START codon). The consensus Kozak sequence varies across organisms in length and nucleotide composition (see for instance [6–8]). However, point mutations in the Kozak sequence affect transcription initiation both in higher [6] and lower [9] eukaryotes.

The consensus Kozak sequence in yeast *S. cerevisiae* was identified by Hamilton et al. [8], who analyzed the translation initiation site (defined as the region between positions −47 and +50) of 99 genes and calculated the frequency at which each of the four nucleotides occupies any of these positions. Position occupancies were also calculated on the subset of highly expressed genes, a main reference point for our work in constructing synthetic 5^′^-UTRs that enhance gene expression.

The portion of 5^′^-UTRs made of 47 nucleotides, taken into account in [8], was rich in adenine and poor in guanine. The abundance of a single nucleotide (adenine) was explained as a means to avoid secondary structures in the leader sequence, which could prevent efficient gene expression. However, this explanation is somewhat challenged by recent in vitro analysis showing a positive correlation between secondary structure in the *S. cerevisiae* 5^′^-UTR and protein abundance [10–12]. The optimal *S. cerevisiae* consensus Kozak sequence (i.e. arising from the group of highly expressed genes) was determined as

$$ \left(\mathrm{A}/\mathrm{T}\right)\mathrm{A}\left(\mathrm{A}/\mathrm{C}\right)\mathrm{A}\left(\mathrm{A}/\mathrm{C}\right)\mathrm{A}\mathbf{ATG}\mathrm{TC}\left(\mathrm{T}/\mathrm{C}\right)\kern2.77626pt , $$

where adenine and thymine at position −6 and adenine and cytosine at position −4 showed the same frequency, whereas at position −2 the frequency of adenine was higher than that of cytosine (50 *%* versus 33 *%*). Interestingly, adenine was always present at position −3. The triplet at positions −2 to −4 exhibits some analogies with the consensus Kozak sequence in mammalian cells. Here, a cytosine can be found at positions −4 and −2 and a purine (A or G) is highly conserved at position −3.

Following the analysis by Hamilton and co-authors [8], we can define those nucleotides whose frequency at a given position is >50 *%* as *strongly conserved*. Accordingly, adenine is strongly conserved at positions −1, −3, −5, −7, and −8. From position −9 to −15, no nucleotide is strongly conserved. In all these positions the most frequent nucleotide is adenine, apart from position −14 where thymine prevails slightly (39 *%* thymine versus 33 *%* adenine). At positions −16 to −20 adenine again becomes strongly conserved. More upstream, short traits where adenine is strongly conserved interrupt longer regions where the frequency of no nucleotide reaches 51 *%*.

A different study on the effect of the 5^′^-UTR on gene expression in *S. cerevisiae* was carried out by Dvir et al. [9]. They generated a pool of 2041 distinct leader sequences by performing random mutations in the −10…−1 region of the *RPL8A* gene (the whole *RPL8A* 5^′^-UTR is only 17 nucleotides long). The *RPL8A* promoter was placed in front of a reporter protein and the influence of diverse 5^′^-UTRs on gene expression was quantified by fluorescence measurements. The data collected in that study showed that protein synthesis is highly influenced by the nucleotide at position −3: a purine (principally adenine) increases protein expression, whereas a pyrimidine lowers it. A further enhancement in gene expression arises when the purine at position −3 is accompanied by other adenines at positions −1 to −4. Moreover, an adenine at position −1 is sufficient to increase protein expression no matter which nucleotide is present at position −3. In contrast, a guanine at position −2 and a cytosine at position −1 have a negative impact on translation initiation. Cytosine is also moderately under-represented at position −2 among the highly expressed *RPL8A* variants.

Taking our cue from the works by Hamilton et al. [8] and Dvir et al. [9], we carried out a detailed study on how synthetic 5^′^-UTRs can alter gene expression in *S. cerevisiae*. By following an approach similar to that in [9], we focused on a unique gene (*CYC1*) and built 58 synthetic variants of the minimal *CYC1* promoter (pCYC1min) via single and multiple point mutations between positions −15 and −1. The starting point of our work was, however, not the original *CYC1* leader sequence but a synthetic one optimized for high gene expression, according to the results in [8]. We fused each synthetic 5^′^-UTR to the CDS of a green fluorescent protein and quantified gene expression via FACS experiments. Our results indicate clearly that nucleotides upstream of the Kozak sequence (positions −9 to −15) play an important role in protein synthesis. Above all, single point mutations, on an adenine background, tend to increase protein expression, whereas multiple mutations to guanine are negatively correlated to gene expression, even when followed by a Kozak sequence containing only adenines. The latter result is due to changes in the mRNA secondary structure as indicated by the results of simulations with the software RNAfold [13, 14]. Our work paves the way for the characterization of eukaryotic promoters through their translational strength.

## Results and discussion

We chose the 5^′^-UTR of the well-studied *S. cerevisiae* *CYC1* promoter [15, 16]. We fused pCYC1min (starting at position −143) to a yeast-enhanced green fluorescent protein (yEGFP) [17] and the *CYC1* terminator. Compared to the complete *CYC1* promoter, pCYC1min contains two of the three TATA boxes and no upstream activating sequences. pCYC1min is a moderately weak promoter and, for this reason, appears to be an ideal candidate for detecting both positive and negative effects of point mutations in the leader sequence on the expression of the downstream reporter protein. The *CYC1* promoter 5^′^-UTR is 71 nucleotides long.

In the following analysis, we refer to the portion of *CYC1* 5^′^-UTR at position −1 to −8 as the *extended Kozak sequence* and that at −9 to −15 as the *upstream region*. In the extended Kozak sequence adenine is strongly conserved in five positions, whereas in the upstream region no nucleotide is strongly conserved. However, adenine is the most frequent at almost every site (see Background).

### The extended Kozak sequence

The original *CYC1* sequence from positions −15 to −1 is CACACTAAATTAATA (hereafter referred to as *k*_0_). According to Dvir et al. [9], the presence of an adenine at positions −1, −3, and −4, together with the absence of guanine at position −2, should make this leader sequence almost optimal for high expression. However, thymine at position −2 and cytosine at position −13 have a frequency lower than 20 *%* and 10 *%*, respectively, among highly expressed *S. cerevisiae* genes [8]. We built our first synthetic *CYC1* leader sequence (*k*_1_) by placing an adenine at each position from −1 to −15.

The fluorescence level associated with *k*_1_ was 6.5 *%* higher than that measured with *k*_0_. However, no statistically significant difference arose from the data gathered on these two leader sequences (*p*-value =0.13). We kept *k*_1_ (the optimized leader sequence) as a template for our next synthetic constructs and built 57 more synthetic 5^′^-UTRs by mutating single or multiple nucleotides in *k*_1_.

The first group of synthetic leader sequences was made by a single point mutation from position −1 to position −8 (see Table 1). Hence, we modified the extended Kozak sequence only, whereas the upstream region was kept in an optimized configuration for high gene expression with adenines at positions −9 to −15.

**Table 1:** Synthetic *CYC1* 5^′^-UTR terminal sequences from *k*_1_ to *k*_25_

ID	Mutation at	Sequence
*k*_0_	-	CACACTAAATTAATA
*k*_1_	-	AAAAAAAAAAAAAAA
*k*_2_		AAAAAAAAAAAAAA**C**
*k*_3_	−1	AAAAAAAAAAAAAA**T**
*k*_4_		AAAAAAAAAAAAAA**G**
*k*_5_		AAAAAAAAAAAAA**C**A
*k*_6_	−2	AAAAAAAAAAAAA**T**A
*k*_7_		AAAAAAAAAAAAA**G**A
*k*_8_		AAAAAAAAAAAA**C**AA
*k*_9_	−3	AAAAAAAAAAAA**T**AA
*k*_10_		AAAAAAAAAAAA**G**AA
*k*_11_		AAAAAAAAAAA**C**AAA
*k*_12_	−4	AAAAAAAAAAA**T**AAA
*k*_13_		AAAAAAAAAAA**G**AAA
*k*_14_		AAAAAAAAAA**C**AAAA
*k*_15_	−5	AAAAAAAAAA**T**AAAA
*k*_16_		AAAAAAAAAA**G**AAAA
*k*_17_		AAAAAAAAA**C**AAAAA
*k*_18_	−6	AAAAAAAAA**T**AAAAA
*k*_19_		AAAAAAAAA**G**AAAAA
*k*_20_		AAAAAAAA**C**AAAAAA
*k*_21_	−7	AAAAAAAA**T**AAAAAA
*k*_22_		AAAAAAAA**G**AAAAAA
*k*_23_		AAAAAAA**C**AAAAAAA
*k*_24_	−8	AAAAAAA**T**AAAAAAA
*k*_25_		AAAAAAA**G**AAAAAAA

First column, 5^′^-UTR identifiers. Second column, position of single point mutations with respect to *k*_1_. Third column, bold font represents mutated nucleotides. *k*_0_ represents the original *CYC1* 5^′^-UTR terminal sequence

The highest fluorescence was recorded for *k*_16_ (where a guanine substituted the adenine at position −5) and the lowest by *k*_9_ (where a thymine replaced the adenine at position −3). Moreover, the fluorescence level of *k*_16_ was statistically significantly different from that of *k*_0_ and *k*_1_. An enhancement in fluorescence due to a guanine at position −5 was a surprising result because guanine is the least frequent nucleotide in yeast *S. cerevisiae* leader sequences. Moreover, no guanine was ever detected at this position among highly expressed genes [8] or provoked any fluorescence enhancement in the work by Dvir et al. [9].

**Fig. 1:**
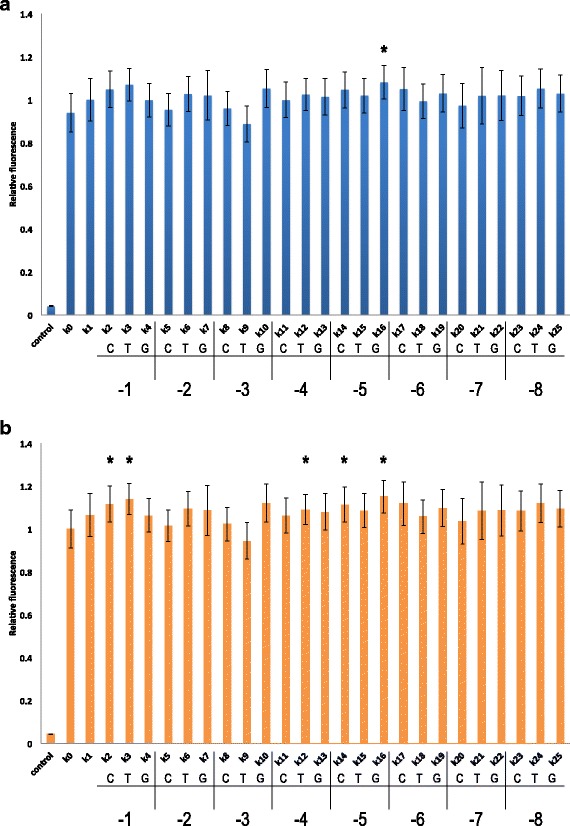
Effect of point mutations in the extended Kozak sequence on fluorescence expression. Fluorescence levels are plotted relative to *k*_1_ (**a**) and *k*_0_ (**b**). Control corresponds to a yeast strain without the yEGFP gene. The nucleotide that replaced an adenine in *k*_1_ and the position at which the mutation took place are given below the name of each synthetic leader sequence. Asterisks, *p*-value <0.05 vs. *k*_1_ (**a**) or *k*_0_ (**b**)

Despite the absence of a statistically significant difference from *k*_1_, the only constructs other than *k*_16_ that resulted in an increase of >5 *%* on the fluorescence level of *k*_1_ were *k*_3_, *k*_10_, and *k*_24_. In particular, in *k*_3_, a thymine replaced an adenine at position −1, and in *k*_10_ the adenine at position −3 was mutated into a guanine. As reported above, adenine at positions −1 and −3 should guarantee high gene expression. Nevertheless, on such an adenine background, less frequent nucleotides at positions −1 or −3 seem to be required to further enhance gene expression. In contrast, a thymine instead of an adenine at position −3 (*k*_9_) was the only mutation that induced a >5 *%* reduction in *k*_1_ fluorescence level. This result is consistent with the observation in [9] that a thymine at position −3 is abundant in poorly expressed genes (Fig. 1 a).

With respect to *k*_0_, all 25 new synthetic leader sequences contained between six and eight mutations. Apart from *k*_9_, all synthetic 5^′^-UTRs showed a fluorescence level higher than that of *k*_0_, five of which were significantly higher. These included positions −1, −4, and −5. As already noted in the comparison with *k*_1_, an adenine just upstream of the START codon seemed to be of no particular advantage for gene expression. Here, a cytosine and a thymine (*k*_2_ and *k*_3_, respectively) performed much better than an adenine. However, with respect to *k*_0_, there were seven more point mutations upstream. At position −4 a thymine (*k*_12_) resulted in the highest fluorescence increment, whereas at position −5, both a cytosine (*k*_14_) and a guanine (*k*_16_) enhanced fluorescence to >10 *%* above that of *k*_0_. Since *k*_0_ has a thymine at positions −2, −5, and −6, each of the five synthetic 5^′^-UTRs that showed statistically significant differences from *k*_0_ were affected by a point mutation at two or more adjacent sites. Three more synthetic leader sequences (*k*_10_,*k*_17_, and *k*_24_) caused a >10 *%* increase in fluorescence compared to *k*_0_, though these differences were not significant (*p*-value >0.05). *k*_10_ and *k*_17_ also had double point mutations at adjacent sites (Fig. 1 b).

### Multiple mutations to guanine

The analysis of our first 25 synthetic 5^′^-UTR sequences gave the surprising result that a single point mutation to guanine—which is essentially absent from the extended Kozak sequence of highly expressed *S. cerevisiae* genes—can enhance the fluorescence level of *k*_1_, a leader sequence optimized for gene expression. Moreover, five of our synthetic 5^′^-UTRs unambiguously (>9 *%*) increased the fluorescence level associated with pCYC1min.

According to our data, a single mutation to guanine can enhance gene expression. However, two previous papers [18, 19] reported that multiple guanines placed in front of a START codon would considerably reduce protein synthesis. Therefore, we assessed how multiple point mutations to guanine affected the translation efficiency of pCYC1min, to determine if they could be used to modulate gene expression.

According to [8], among highly expressed *S. cerevisiae* genes, guanine is the least frequent nucleotide between positions −1 and −15, with the exception of position −7, in which the least frequent nucleotide is cytosine. We constructed a synthetic 5^′^-UTR that reflects this sequence (*k*_26_; Table 2). This shut down gene expression, as shown by the corresponding fluorescence level not being significantly different (*p*-value =0.21) from our negative control (an *S. cerevisiae* strain that did not contain the yEGFP gene).

**Table 2:** Synthetic *CYC1* 5^′^-UTR terminal sequences from *k*_26_ to *k*_38_

ID	MFE (kcal/mol)	Sequence
*k*_0_	**-241.21**	CACACTAAATTAATA
*k*_1_	**-241.21**	AAAAAAAAAAAAAAA
*k*_26_	−261.39	**GGGGGGGGCGGGGGG**
*k*_27_	−247.04	AAAAAAA**GCGGGGGG**
*k*_28_	−246.41	**GGGGGGG**AAAAAAAA
*k*_29_	−245.97	AAAAAAA**GCGGGGG**A
*k*_30_	−244.28	AAAAAAA**GCGGGG**A**G**
*k*_31_	−247.46	AAAAAAA**GCGGG**A**GG**
*k*_32_	**-241.21**	**GGGG**AAAAAAAAAAA
*k*_33_	−241.93	**G**A**G**A**G**A**G**AAAAAAAA
*k*_34_	−246.59	**GGGGGG**AAAAAAAAA
*k*_35_	−242.5	**GGGGG**AAAAAAAAAA
*k*_36_	**-241.21**	**GGG**AAAAAAAAAAAA
*k*_37_	**-241.21**	**GG**AAAAAAAAAAAAA
*k*_38_	**-241.21**	**G**AAAAAAAAAAAAAA

Nucleotides in bold are mutations with respect to *k*_1_ sequence. *k*_0_ is shown for comparison. MFE, minimum free energy, computed in RNAfold

We tested whether multiple mutations to guanine (cytosine at position −7) would affect gene expression in a different way when they covered either the whole extended Kozak sequence (*k*_27_) or the upstream region (*k*_28_). Since mutations were made with respect to *k*_1_, all the non-mutated sites contained an adenine. Surprisingly, we found that the two configurations were equivalent for gene expression (*p*-value >0.40) and reduced *k*_1_ fluorescence level by about half.

Starting from *k*_27_, we replaced the guanine at positions −1 (*k*_29_), −2 (*k*_30_), and −3 (*k*_31_) with an adenine to determine whether a single adenine at the three positions just upstream of the START codon would enhance fluorescence expression when the other sites of the extended Kozak sequence were occupied either by a guanine or a cytosine. At position −1 an adenine showed no improvement on the fluorescence of *k*_27_. Interestingly, at positions −2 and −3, an adenine caused a drop in gene expression to approximately 7 *%* of the *k*_1_ fluorescence level. These results demonstrate that an adenine *per se* cannot improve gene expression even when it occupies position −3 or −1. More generally, we can conclude that the effect on gene expression of a single point mutation in the leader sequence is strongly context-dependent.

**Fig. 2:**
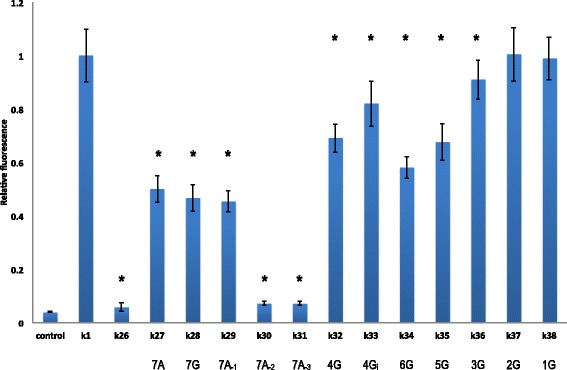
Multiple point mutations to guanine. The ratio between the fluorescence level of the synthetic 5^′^-UTRs from *k*_26_ to *k*_38_ and that of *k*_1_ are reported. The number of adenines or guanines in the upstream region is given below the leader sequence name (from *k*_27_ to *k*_38_). The subscripts −1, −2, and −3 indicate that an adenine is present in the extended Kozak sequence only at the corresponding position. Subscript *i* represents *intermixed* (see main text). Asterisks, *p*-value <0.05 vs. *k*_1_

Finally, to understand better how important the upstream region is for gene expression, we progressively reduced the number of guanines from seven (*k*_28_) to one (*k*_38_). Starting from position −9, we replaced a guanine with an adenine at each step and saw that the fluorescence level increased almost linearly with the number of adenines (Fig. 2 and Additional file 1). The last sequence in which the fluorescence level was statistically significantly different from that of *k*_1_ was *k*_36_, in which guanines were present at positions −13 to −15. A guanine alone at position −15 or accompanied by another at position −14 did not result in a significant difference in fluorescence level from that of *k*_1_. Therefore, even in the presence of an extended Kozak sequence optimized for high gene expression, multiple mutations in the upstream region have evident repercussions on protein synthesis and can be used as a means of tuning protein abundance. An explanation for this result is presented in the Computational Analysis section, below. Interestingly, four guanines intermixed with adenines (*k*_33_) in the upstream region reduced *k*_1_ fluorescence to a smaller extent than four guanines in a row (*k*_32_), providing further confirmation that the effect on gene expression of point mutations inside the 5^′^-UTR is highly dependent on the nucleotidic context (Fig. 2; see Additional file 1 for a comparison with *k*_0_ fluorescence).

### The upstream region

The previous analysis confirmed that the effect on gene expression due to both single and multiple mutations within the 5^′^-UTR is strongly context-dependent. Moreover, our data clearly showed that changes not only in the Kozak sequence but also inside the upstream region markedly affect gene expression. We therefore performed point mutations on *k*_1_ between positions −9 and −15 (Table 3) to assess whether a single nucleotide different from adenine can change the translation rate when placed into the upstream region.

**Table 3:** Synthetic *CYC1* 5^′^-UTR terminal sequences from *k*_39_ to *k*_58_

ID	Mutation at	Sequence
*k*_0_	-	CACACTAAATTAATA
*k*_1_	-	AAAAAAAAAAAAAAA
*k*_39_		AAAAAA**C**AAAAAAAA
*k*_40_	−9	AAAAAA**T**AAAAAAAA
*k*_41_		AAAAAA**G**AAAAAAAA
*k*_42_		AAAAA**C**AAAAAAAAA
*k*_43_	−10	AAAAA**T**AAAAAAAAA
*k*_44_		AAAAA**G**AAAAAAAAA
*k*_45_		AAAA**C**AAAAAAAAAA
*k*_46_	−11	AAAA**T**AAAAAAAAAA
*k*_47_		AAAA**G**AAAAAAAAAA
*k*_48_		AAA**C**AAAAAAAAAAA
*k*_49_	−12	AAA**T**AAAAAAAAAAA
*k*_50_		AAA**G**AAAAAAAAAAA
*k*_51_		AA**C**AAAAAAAAAAAA
*k*_52_	−13	AA**T**AAAAAAAAAAAA
*k*_53_		AA**G**AAAAAAAAAAAA
*k*_54_		A**C**AAAAAAAAAAAAA
*k*_55_	−14	A**T**AAAAAAAAAAAAA
*k*_56_		A**G**AAAAAAAAAAAAA
*k*_57_		**C**AAAAAAAAAAAAAA
*k*_58_	−15	**T**AAAAAAAAAAAAAA
*k*_38_		**G**AAAAAAAAAAAAAA

As in Table 1, the position of single point mutations with respect to *k*_1_ is given in the second column, whereas the mutated nucleotides are written in bold in the third column. Also reported are *k*_0_ and *k*_38_, the latter initially used to study the effect of guanines in the upstream region

All point mutations (except the one in *k*_38_) resulted in a fluorescence level higher than that associated with *k*_1_. Notably, in eight cases, the increase in fluorescence was statistically significant (>10 *%* higher than *k*_1_ fluorescence). These eight mutations included four contiguous positions, from −11 to −14. None of these were taken into account in the reference work by Dvir et al. [9].

At position −11, a guanine instead of an adenine (*k*_47_) enhanced fluorescence expression by >15 *%*, whereas cytosine and thymine had no significant effects. Every mutation at position −12 increased the fluorescence of *k*_1_. The greatest change (>15 *%*) was due to a guanine (*k*_50_). Mutations at position −13 also strongly enhanced *k*_1_ fluorescence level. Two point mutations—cytosine (*k*_51_) and guanine (*k*_53_)—resulted in statistically significant differences in fluorescence from *k*_1_, whereas a thymine (*k*_52_) augmented *k*_1_ fluorescence by about 14 *%* but this did not reach statistical significance. It should be noted that among all our 58 synthetic 5^′^-UTRs, *k*_51_ had the highest fluorescence level—almost 17 *%* higher than that of *k*_1_.

**Fig. 3:**
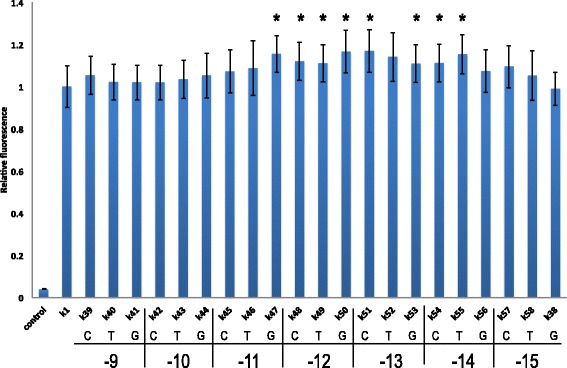
Effect of point mutations in the upstream region on fluorescence relative to *k*_1_. The nucleotide that replaced an adenine in *k*_1_ and the position at which the mutation took place are given below the name of each synthetic leader sequence. Asterisks, *p*-value <0.05 vs. *k*_1_

Finally, two different point mutations at position −14 led to an increase in fluorescence: a cytosine (*k*_54_) and a thymine (*k*_55_) (Fig. 3; see Additional file 1 for a comparison with *k*_0_).

Together, the results of this last analysis of the upstream region underline another surprising result: single point mutations upstream of the Kozak sequence, in particular at positions −12 and −13, were those that most enhanced gene expression from a context rich in adenines.

### Computational analysis

We carried out simulations with RNAfold to investigate possible correlations between computed mRNA secondary structures, together with their corresponding minimum free energies (MFEs), and measured fluorescence levels. Our analysis provides an explanation for the drop in fluorescence due to multiple mutations from adenine to guanine (and cytosine) in the −15…−1 region. In contrast, no plausible justification for the effects of single point mutations on translational efficiency emerged from simulations with RNAfold.

As an input for RNAfold, we used mRNA sequences starting at the transcription start site of pCYC1min [16] and ending at the poly-A site of the *CYC1* terminator [20]. Each sequence was 937 nucleotides long. From preliminary simulations, we observed that a poly-A chain with a variable length of 150–200 nucleotides had no significant effect on mRNA folding. All mRNA secondary structures were calculated at 30 °C (the temperature at which we grew *S. cerevisiae* cells for the FACS experiments).

**Fig. 4:**
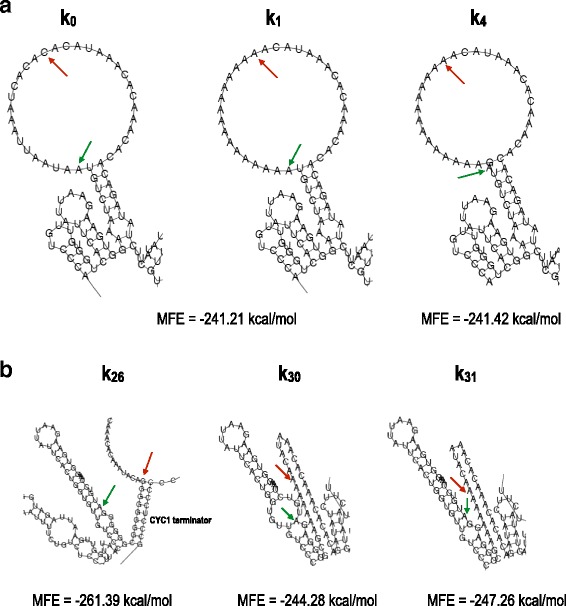
mRNA secondary structures. **a** A *giant hairpin* is present in the mRNA secondary structure corresponding to the MFE of both *k*_0_ and *k*_1_. The hairpin loop contains the −15…−1 region. The portion of the 5^′^-UTR in our analysis is free from any pairing interactions in its wild-type configuration (*k*_0_) and in that theoretically optimized for high protein expression (*k*_1_). The loop of the giant hairpin is reduced in *k*_4_ owing to the base-pairing interaction between the guanine at position −1 and the cytosine at position −31. In every mRNA structure presented, a green arrow indicates position +1, and a red arrow indicates position −15. **b** The disruption of the giant hairpin induces a decrease in the MFE of the mRNA secondary structure. *k*_26_ and *k*_31_ are associated with the lowest MFEs computed in our analysis. The two sequences contain multiple guanines in the extended Kozak sequence involved in pairing interactions with the CDS. A similar pattern is also present in *k*_30_. Here, however, a second mini-loop around the START codon provokes an increase in MFE. The MFE of *k*_26_ is substantially lower than those of *k*_30_ and *k*_31_ because of the presence of another stem due to pairing interactions between the upstream region and the *CYC1* terminator. Nevertheless, the fluorescence levels of *k*_30_ and *k*_31_ are only approximately 1.2-fold higher than that of *k*_26_

*k*_0_ and *k*_1_ have the same MFE: −241.21 kcal/mol. This is the highest—and the most common—within the collection of 59 sequences analyzed in this work (see Additional file 1). The mRNA secondary structure corresponding to this MFE is characterized by the presence of a *giant hairpin* between positions −40 and +10. The hairpin loop goes from position −31 to position +1 and contains the whole 5^′^-UTR portion we have targeted here. The hairpin stem is made of nine base-pairs, of which only one gave a “mismatch” because of an adenine at position −38 and +8 (see Fig. 4 a).

**Fig. 5:**
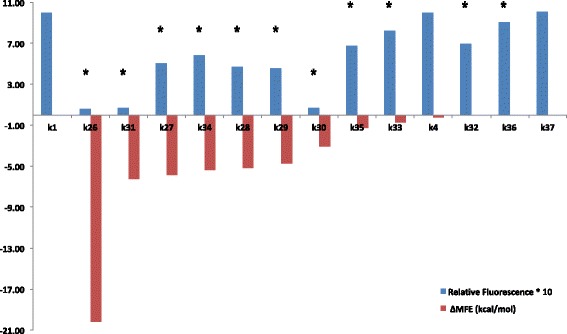
Low MFE values are associated with reduced fluorescence expression. *Red bars*, difference between MFEs of the corresponding 5^′^-UTR and *k*_1_ (*Δ*MFE). *Blue bars*, 10-fold magnified ratio between the fluorescence level of the indicated 5^′^-UTR and that of *k*_1_. Apart from *k*_1_, sequences are sorted by increasing *Δ*MFE. All sequences except *k*_4_ contain multiple point mutations with respect to *k*_1_. Asterisks above *blue bars*, *p*-value <0.05 vs. *k*_1_

Multiple mutations to guanines either in the upstream region or the extended Kozak sequence originate base-pairing interactions between, at least, a portion of the −15…−1 region and the CDS (yEGFP) or the *CYC1* terminator. As a consequence, the giant hairpin is destroyed and replaced by one or two stems that lower the MFE of the mRNA secondary structure (Table 2). Most of the MFE values smaller than −241.21 kcal/mol were associated with fluorescence levels lower than that of *k*_1_ (Fig. 5). This result is in agreement with the notion, supported also by [8, 9], that stable mRNA secondary structures in the 5^′^-UTR reduce protein expression. However, the fluorescence levels we measured did not increase proportionally to increments in the MFE. Moreover, in two cases (*k*_32_ and *k*_36_) RNAfold predicted a giant hairpin in the mRNA structure, whereas the fluorescence levels from our experiments were significantly lower than that of *k*_1_ (Fig. 5 and Additional file 1).

*k*_26_ was designed by choosing the least frequent nucleotides between positions −15 and −1 among a set of highly expressed *S. cerevisiae* genes. The corresponding MFE (−261.39 kcal/mol) was the lowest within the ensemble of transcription units considered in this work. No giant hairpin was present in the MFE mRNA secondary structure as the −15…−1 region was sequestered into two different stems. The guanines between positions −1 and −6 were part of a long stem and paired with a hexamer at the beginning of the yEGFP sequence (positions +33 to +38). In contrast, positions −9 to −15 paired with a region of the *CYC1* terminator, at positions +750 to +758 (Fig. 4 b).

A fluorescence level just above that of *k*_26_ was registered for *k*_30_ and *k*_31_. Both differed from *k*_26_ for the upstream region (made of seven adenines) and the presence of an adenine in the extended Kozak region (at positions −2 and −3, respectively). Similarly to *k*_26_, the first five nucleotides of the extended Kozak region of *k*_30_ and the first six of *k*_31_ were sequestered into a stem with the CDS. However, differently from *k*_26_, the upstream regions of *k*_30_ and *k*_31_ were entirely free from any pairing interactions (see Fig. 4 b). Their MFEs (−244.28 and −247.26 kcal/mol, respectively) were also significantly higher than that of *k*_26_. These three sequences suggest that a condition for markedly lowering protein expression is to enclose the nucleotides at positions −1 to −5 in an mRNA secondary structure. Moreover, not all of these nucleotides have to participate in base-pairing interactions. Indeed, a guanine at position −1 (*k*_30_) or −2 (*k*_26_ and *k*_31_) is “free” and responsible for the presence of a mini-loop in the mRNA structure.

**Fig. 6:**
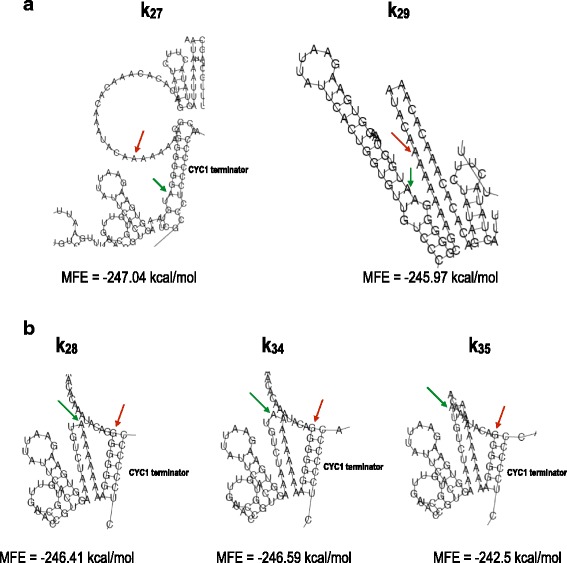
mRNA secondary structures. **a** *k*_27_ differs from *k*_29_ only by a guanine instead of an adenine at position −1. However, their mRNA secondary structures are dissimilar. In *k*_27_, the extended Kozak sequence is involved in base-pairing interactions with the *CYC1* terminator, whereas in *k*_29_ the extended Kozak sequence is locked into a stem with the CDS. The MFE associated with *k*_27_ is lower than that of *k*_29_, but there is no difference between the fluorescence levels of the two sequences (*p*-value =0.20). **b** Multiple guanines in the upstream region give rise to mRNA structures characterized by base-pairing interactions between the 5^′^-UTR and the *CYC1* terminator. *k*_28_ and *k*_34_ have six guanines in a stem with the *CYC1* terminator, whereas *k*_35_ has only 5 guanines in an analogous structure. This causes an increase in MFE and consequently a higher fluorescence

However, this hypothesis is contradicted by *k*_29_. The MFE of this sequence (−245.97 kcal/mol) is comparable to that of *k*_30_ and *k*_31_, and the corresponding mRNA secondary structure is very similar to that of *k*_31_ (Fig. 6 a). Nevertheless, the fluorescence level associated with *k*_29_ was more than 6-fold higher than that of *k*_31_ and amounted to 45% of that of *k*_1_.

*k*_27_ shared with *k*_29_– *k*_31_ an upstream region made only of adenines. However, unlike in these three sequences, the extended Kozak sequence of *k*_27_ did not contain any adenine. The MFE of *k*_27_ (−247.04 kcal/mol) was comparable to that of *k*_29_– *k*_31_, but its corresponding mRNA secondary structure had a different configuration. Indeed, all nucleotides of the extended Kozak sequence (with the exception of the cytosine at position −7) were involved in base-pairing interaction not with the CDS but with the *CYC1* terminator (positions +755 to +762; Fig. 6 a). The fluorescence level of *k*_27_ was slightly higher than that of *k*_29_, i.e. almost 7-fold greater than that of *k*_31_.

The five sequences considered so far (*k*_26_, *k*_27_, *k*_29_– *k*_31_) have in common an extended Kozak region rich in guanine that was sequestered into a stem in the MFE mRNA secondary structure. In four cases, the extended Kozak sequence paired (partially) with the CDS, and in one case (*k*_27_) with the *CYC1* terminator. The MFE of *k*_26_ was the lowest, as its upstream region was also sequestered into a stem. The other four sequences showed very similar MFE values but rather different fluorescence levels.

The other group of sequences affected by multiple mutations with respect to *k*_1_ had only adenines in the extended Kozak sequence and a variable number of guanines in the upstream region.

*k*_28_, *k*_34_, and *k*_35_ had, respectively, 7, 6, and 5 guanines in a row from position −15 downstream. Although the MFE of *k*_35_ was clearly higher than that of *k*_28_ and *k*_34_ (Table 2), the three sequences gave rise to similar mRNA structures where at least five guanines of the upstream region (plus the first adenine downstream) were locked into a stem due to base-pairing interactions with the *CYC1* terminator (see Fig. 6 b).

Interestingly, both the MFE and fluorescence level of *k*_28_ were comparable to those of *k*_27_ and *k*_29_. Hence, even if the Kozak sequence was free of pairing interactions, the sequestering of the upstream region into a stem was enough to guarantee a clear drop in protein expression. This is further confirmation of the role played by the nucleotides upstream of the Kozak sequence in tuning protein expression.

A different MFE mRNA secondary structure was obtained for *k*_33_ (four guanines, intermixed with adenines), in which half of the extended Kozak sequence and almost the whole upstream region were involved in base-pairing interactions with the CDS, giving rise to a long stem. However, compared to *k*_35_, where only five nucleotides of the upstream region were locked into a stem with the *CYC1* terminator, *k*_33_ showed a higher MFE as well as a higher fluorescence level (Fig. 5 and Additional file 1).

Finally, for *k*_32_, *k*_36_, and *k*_37_ (with four, three, and two guanines in the upstream region, respectively) RNAfold returned the same MFE as for *k*_1_. The corresponding mRNA secondary structures were all characterized by the presence of the the giant hairpin (see Additional file 1). Compared to our experimental data, this result was plausible only for *k*_37_ but in apparent disagreement with the measurements for *k*_32_ and *k*_36_, whose fluorescence levels were significantly lower than that of *k*_1_ (Fig. 5). In particular, the fluorescence of *k*_32_ only corresponded to about 69% of that of *k*_1_. Therefore, it can be argued that in vivo *k*_32_ and *k*_1_ share the same MFE and mRNA secondary structure, as suggested by the in silico simulations.

In contrast to the multiple point mutations, of the single point mutations on *k*_1_, only *k*_4_ caused a modification in the structure of the giant hairpin and a consequent decrease in the MFE. *k*_4_ carries a guanine at position −1 that pairs with the cytosine at position −31 such that the length of the loop is reduced from 32 to 29 nucleotides and the MFE is lowered to −241.42 kcal/mol (Fig. 4 a). According to our data, this minimal change has no effect on fluorescence expression. All the other point mutations that induced a fluorescence level significantly higher than that of *k*_1_ (namely, *k*_16_, *k*_47_– *k*_51_, and *k*_53_– *k*_55_) were characterized by the same MFE and corresponding mRNA secondary structure as *k*_1_, according to the RNAfold simulations.

## Conclusions

To date, the role played by 5^′^-UTR in eukaryotic cell transcription initiation has not been studied deeply nor clearly understood. As for *S. cerevisiae*, two main works in the literature [8, 9] showed that the 5^′^-UTR is rich in adenine and poor in guanine, mainly in the proximity of the START codon. The consensus Kozak sequence, determined on highly expressed genes, shows a strongly conserved adenine (frequency >50 *%*) at positions −1, −3, and −5, whereas cytosines at positions −2 and −4 and a thymine at position −6 are also strongly present. Adenines at positions −3 and −1 are suggested to be necessary for enhancing gene expression. In contrast, two other works [18, 19] showed that several adjacent guanines placed just upstream of the START codon induced a significant drop in protein synthesis.

We analyzed the 5^′^-UTR of the constitutive *S. cerevisiae* *CYC1* promoter. We fused pCYC1min to a reporter protein and quantified the strength of 58 synthetic leader sequences using fluorescence measurements. We took into account only the 15 nucleotides just upstream of the START codon. In a previous report, Dvir et al. [9] examined positions −1 to −10 of a different *S. cerevisiae* gene, *RPL8A*.

Our starting point was the construction of a synthetic 5^′^-UTR where each position from −1 to −15 was taken by an adenine—the nucleotide that seems the most favorable for high gene expression. We called this sequence *k*_1_. With respect to the original *CYC1* 5^′^-UTR (here termed *k*_0_), *k*_1_ did not represent a significant improvement. In contrast, a statistically significant enhancement in fluorescence was achieved by single point mutations in *k*_1_. Surprisingly, a cytosine and a thymine in position −1 proved to be more efficient than an adenine, which disagrees with previous claims that an adenine at position −1 is on its own sufficient for enhancing gene expression. Furthermore, point mutations at positions −4 and −5 also showed a statistically significant improvement with respect to the fluorescence of *k*_0_. In particular, a guanine (the least frequent nucleotide in *S. cerevisiae* leader sequences) at position −5 resulted in a statistically significant increase in fluorescence, even compared to *k*_1_. Hence, single point mutations on an adenine background seem to constitute a novel technique for improving translational strength.

We also studied the effects that multiple mutations to guanine can have on gene expression. Here, the starting point was a leader sequence containing the least frequent nucleotides in highly expressed *S. cerevisiae* genes [8] at positions −1 to −15. They were all guanines apart from a cytosine at position −7. This sequence, here termed *k*_26_, switched off fluorescence expression. By mutating only the extended Kozak sequence (positions −1 to −8) or the upstream region (positions −9 to −15), we obtained almost identical results, namely about half of the fluorescence expressed by *k*_1_. This was the first hint that mutations in the upstream region (or, more generally, outside the Kozak sequence) can markedly affect gene expression. Furthermore, we showed that gene expression can be tuned just by varying the number of adjacent guanines in the upstream region while keeping the extended Kozak sequence made of adenines only. This represents another possible approach to engineering synthetic promoters that differ in their translational strength. We also noticed that four guanines intermixed with adenines reduce translation initiation less than four guanines in a row. This confirms that the effect of point mutations (multiple or single) on gene expression is highly context-dependent.

Simulations with RNAfold led to an explanation for the changes in fluorescence expression observed in the presence of multiple mutations from adenine to guanine (and cytosine at position −7). The *k*_1_ mRNA sequence folds into an MFE secondary structure characterized by a giant hairpin whose loop contains the entire −15…−1 region. Therefore, in this configuration, both the extended Kozak sequence and the upstream region are free from base-pairing interactions. Moreover, this mRNA secondary structure returns the highest MFE among the 59 sequences analyzed in this work and seems to foster protein synthesis. Multiple mutations to guanine (and cytosine) cause either the extended Kozak sequence or the upstream region to be locked into a stem due to base-pairing interactions with the CDS or the *CYC1* terminator. As a result, the giant hairpin is destroyed, the MFE is lowered, and fluorescence expression is decreased to different extents. Although it was not possible to identify the precise relationship between MFEs and fluorescence levels, our results mostly agree with the notion that stable mRNA structures in the 5^′^-UTR hinder protein expression.

Finally, we carried out single point mutations on the sole upstream region of *k*_1_ and determined whether positions “far” from the START codon played an important role in protein synthesis. We found that eight sequences, with a single point mutation between positions −11 and −14, produced significant increases in *k*_1_ fluorescence level. Position −12 turned out to be the most critical, because each mutation led to significantly greater fluorescence. Moreover, the highest increase in fluorescence—from all 58 synthetic 5^′^-UTRs—was obtained by turning the adenine at position −13 into a cytosine. Therefore, the leader configuration upstream of the Kozak sequence has a marked influence on translation initiation.

As in the reference work by Dvir et al. [9], the main limit in our analysis is that it was carried out on a single gene. Hence, the results shown here might be not completely valid for other *S. cerevisiae* genes. On the whole, our work shows that 5^′^-UTRs can be exploited to generate new libraries of synthetic *S. cerevisiae* promoters. In particular, mutations on the 5^′^-UTR should not be limited to the Kozak sequence but they should also involve upstream non-conserved regions. As in the *CYC1* gene, these modifications might lead to very high increases (or decreases) in translation initiation. However, further studies investigating entire 5^′^-UTRs are advisable. Our simulations with RNAfold showed how, at the mRNA level, interactions between the promoter (5^′^-UTR) and either the CDS or the terminator (3’-UTR) sequence influence gene expression considerably. This notion should be taken into account to improve the modelling of basic modules for eukaryotic gene circuits (as we previously described in [21]). Our hope is that this work will emphasize the fact that part characterization—a fundamental concept in Synthetic Biology—is still far from being achieved and more basic experiments on standard biological parts (and subparts) are required for a comprehensive description of the basic components of synthetic gene circuits. Only with such accurate knowledge could Synthetic Biology be regarded as a proper engineering discipline.

## Methods

### Plasmid construction

Backbones for all the plasmids used in this work were either the yeast integrative shuttle-vector pRSII406 (Addgene-35442, a gift from Steven Haase) [22] or the modified version pMM125, where the BsaI site in the ampicillin resistance gene and the BpiI site in the URA3 marker were removed via silent mutations. The minimal *CYC1* promoter (pCYC1min) was extracted from the yeast *S. cerevisiae* genome (strain FY1679-08A, see below) following the procedure described in [23].

Every transcription unit expresses the yeast enhanced green fluorescent protein (yEGFP) obtained from pRS31-glag [24] (courtesy of the Hasty lab, University of California, San Diego, USA). A slightly different version termed yEGFPgg (where the BsaI site was removed through a silent mutation) was used in the plasmids assembled using the Golden Gate method [25]. The *CYC1* terminator (CYC1t) [26] placed at the end of every transcription unit was obtained from pRS403-pGAL1-strongSC_GFP (Addgene-22316, a gift from David Bartel).

Plasmids containing 5^′^-UTR from *k*_0_ to *k*_38_ were constructed via isothermal assembly [27]. Those containing 5^′^-UTR from *k*_39_ to *k*_58_ were assembled using the Golden Gate method [25]. For this purpose, we built an acceptor vector (pMM247) where 15-nucleotide-long sequences could be inserted between two BsaI cutting sites, namely TACA (i.e. position −19…−16 of pCYC1min) and ATGT (the first four nucleotides of yEGFPgg). Short DNA sequences containing *k*_39_ to *k*_58_ extended with the above BsaI sites were prepared by Comate Bioscience Co., Ltd. (Harbin, China).

To extract DNA sequences from plasmids, we used touchdown PCR. DNA elution from agarose gel was carried out with the QIAGEN-28604 DNA Elution Kit. Isothermal assembly required 1 h at 50 °C. For Golden Gate assembly, the insert (*k*_*i*_ sequences, *i*:39,…,58) and the pMM247 acceptor vector were combined in an equimolar ratio and mixed with a master mix (1 *μ* *l* BsaI 20 units /*μ* *l*, NEB-R0535S; 2 *μ* *l* Cutsmart buffer NEB; 1 *μ* *l* T4 ligase 400 units /*μ* *l*, NEB-M0202S; 2 *μ* *l*10 *mM* ATP, Sigma-Aldrich-A7699) to a final 15 *μ* *l* volume. The thermocycler program was set to three cycles of 10 min at 40 °C and another 10 min at 16 °C. These cycles were followed by 10 min at 50 °C, 20 min at at 80 °C, and the final temperature was set to 16 °C.

*E. coli* competent cells (strain DH5 *α*, Life Technology, 18263-012) transformed with our plasmids (30-s heatshock at 42 °C) were grown overnight at 37 °C in LB (Luria-Bertani) broth or plates (Bacto tryptone 10%, yeast extract 5%, NaCl 10%, agar 15% for the plates) supplied with ampicillin. Plasmid extraction from bacterial cells was carried out using standard methods [28]. All plasmids were sequenced (Sanger method) to check the correctness of the new synthetic constructs.

### Yeast strain construction

Our synthetic plasmids were integrated into the genome of the yeast *S. cerevisiae* strain FY1679-08A (MATa; ura3-52; leu2 *Δ*1; trp1 *Δ*63; his3 *Δ*200; GAL2), Euroscarf (Johann Wolfgang Goethe University, Frankfurt, Germany). Genomic integration was carried out as described in [29]. About 5 *μ* *g* of plasmidic DNA was linearized at the URA3 marker with the restriction enzyme StuI (NEB-R0187S). Transformed cells were grown on plates containing synthetic selective medium (SD-URA; 2% glucose, 2% agar) for about 36 h at 30 °C.

### Flow cytometry

Yeast cells were grown overnight in synthetic complete medium (SDC) at 30 °C. They were diluted in the morning to approximately 1:100 and allowed to grow in SDC for up to 5 h longer, so that their optical density at 600 *nm* wavelength (*OD*_600_) was always between 0.2 and 2.0 (exponential phase). Fluorescence measurements were performed with a BD Accuri C6 cytometer (488 *nm* laser, 533/30 filter). The FACS machine set-up was checked at the beginning and end of each experiment, using fluorescent beads (AlignFlow, Life Technologies-A16500). Reliable measurements were considered as only those where the relative difference between the initial and final values of the peaks of the beads was lower than 5%. Data were analyzed with the flowcore R-Bioconductor package [30]. Fluorescence levels were compared using Welch’s two-sided t-test (*p*-value <0.05 was considered significant) and were estimated as the mean values of at least three independent experiments (carried out on different days and each with 30000 recorded samples). Standard deviations were calculated on these mean values. The error on relative fluorescence values (ratios) was computed using the error propagation formula.

## Additional file

Additional file 1 Supplementary Material. (PDF 474 kb)

## Abbreviations

5’-UTR: 5’untranslated region
CDS: Coding sequence
CYC1t: *CYC1* terminator
FACS: Fluorescence activated cell sorting
LB: Luria-Bertani (medium)
MFE: Minimum free energy
pCYC1min: Minimal *CYC1* promoter
RBS: Ribosome binding site
SDC: Synthetic complete medium
yEGFP: Yeast-enhanced green fluorescent protein
