# Supplementary material for: Nucleotides upstream of the Kozak sequence strongly influence gene expression in the yeast S. cerevisiae
Source: J Biol Eng. 2017 Aug 21;11:25. doi: 10.1186/s13036-017-0068-1 (PMC5563945; doi:10.1186/s13036-017-0068-1)
Supplement: Supplementary file 1 — Supplementary Material. (PDF 474 kb) [file 13036_2017_68_MOESM1_ESM.pdf]

Nucleotides upstream of the Kozak sequence exert high influence on gene expression in yeast *S. cerevisiae*
Supplementary Material

Jing Li[1], Qiang Liang[1], Wenjiang Song[1], and Mario Andrea Marchisio[1,*]

[1]School of Life Science and Technology, Harbin Institute of Technology,

2 Yikuang Street, Nan Gang District, 150080 Harbin, P. R. China

[*]corresponding author

email addresses: JL, 920138462@qq.com; QL, 804257847@qq.com; WS, 1176784021@qq.com

MAM: marchisio@hit.edu.cn, mamarchisio@yahoo.com

Plasmids used in this work

Plasmid name	Construct
pMM61	pRSII406-pCYC1min-yEGFP-CYC1t
pMM75	pRSII406-pCYC1min-k1-yEGFP-CYC1t
pMM76	pRSII406-pCYC1min-k2-yEGFP-CYC1t
pMM121	pRSII406-pCYC1min-k3-yEGFP-CYC1t
pMM113	pRSII406-pCYC1min-k4-yEGFP-CYC1t
pMM77	pRSII406-pCYC1min-k5-yEGFP-CYC1t
pMM78	pRSII406-pCYC1min-k6-yEGFP-CYC1t
pMM79	pRSII406-pCYC1min-k7-yEGFP-CYC1t
pMM83	pRSII406-pCYC1min-k8-yEGFP-CYC1t
pMM84	pRSII406-pCYC1min-k9-yEGFP-CYC1t
pMM85	pRSII406-pCYC1min-k10-yEGFP-CYC1t
pMM86	pRSII406-pCYC1min-k11-yEGFP-CYC1t
pMM93	pRSII406-pCYC1min-k12-yEGFP-CYC1t
pMM130	pRSII406-pCYC1min-k13-yEGFP-CYC1t
pMM97	pRSII406-pCYC1min-k14-yEGFP-CYC1t
pMM98	pRSII406-pCYC1min-k15-yEGFP-CYC1t
pMM99	pRSII406-pCYC1min-k16-yEGFP-CYC1t
pMM100	pRSII406-pCYC1min-k17-yEGFP-CYC1t
pMM116	pRSII406-pCYC1min-k18-yEGFP-CYC1t
pMM101	pRSII406-pCYC1min-k19-yEGFP-CYC1t
pMM104	pRSII406-pCYC1min-k20-yEGFP-CYC1t
pMM105	pRSII406-pCYC1min-k21-yEGFP-CYC1t
pMM106	pRSII406-pCYC1min-k22-yEGFP-CYC1t
pMM107	pRSII406-pCYC1min-k23-yEGFP-CYC1t
pMM108	pRSII406-pCYC1min-k24-yEGFP-CYC1t
pMM118	pRSII406-pCYC1min-k25-yEGFP-CYC1t
pMM143	pRSII406-pCYC1min-k26-yEGFP-CYC1t
pMM141	pRSII406-pCYC1min-k27-yEGFP-CYC1t
pMM150	pRSII406-pCYC1min-k28-yEGFP-CYC1t
pMM151	pRSII406-pCYC1min-k29-yEGFP-CYC1t
pMM153	pRSII406-pCYC1min-k30-yEGFP-CYC1t
pMM154	pRSII406-pCYC1min-k31-yEGFP-CYC1t
pMM166	pRSII406-pCYC1min-k32-yEGFP-CYC1t
pMM167	pRSII406-pCYC1min-k33-yEGFP-CYC1t
pMM175	pRSII406-pCYC1min-k34-yEGFP-CYC1t
pMM159	pRSII406-pCYC1min-k35-yEGFP-CYC1t
pMM168	pRSII406-pCYC1min-k36-yEGFP-CYC1t
pMM158	pRSII406-pCYC1min-k37-yEGFP-CYC1t
pMM176	pRSII406-pCYC1min-k38-yEGFP-CYC1t

Table S1: List of the plasmids assembled with the Gibson method.

Plasmid name	Construct
pMM247	pRSII406-pCYC1min_without_k0-(TACA)**BsaI**–**BsaI**(ATGT)-yEGFPgg-CYC1t
pMM272	pMM247-pCYC1min-k39-yEGFPgg-CYC1t
pMM273	pMM247-pCYC1min-k40-yEGFPgg-CYC1t
pMM274	pMM247-pCYC1min-k41-yEGFPgg-CYC1t
pMM275	pMM247-pCYC1min-k42-yEGFPgg-CYC1t
pMM276	pMM247-pCYC1min-k43-yEGFPgg-CYC1t
pMM277	pMM247-pCYC1min-k44-yEGFPgg-CYC1t
pMM303	pMM247-pCYC1min-k45-yEGFPgg-CYC1t
pMM304	pMM247-pCYC1min-k46-yEGFPgg-CYC1t
pMM305	pMM247-pCYC1min-k47-yEGFPgg-CYC1t
pMM306	pMM247-pCYC1min-k48-yEGFPgg-CYC1t
pMM307	pMM247-pCYC1min-k49-yEGFPgg-CYC1t
pMM309	pMM247-pCYC1min-k50-yEGFPgg-CYC1t
pMM310	pMM247-pCYC1min-k51-yEGFPgg-CYC1t
pMM311	pMM247-pCYC1min-k52-yEGFPgg-CYC1t
pMM312	pMM247-pCYC1min-k53-yEGFPgg-CYC1t
pMM321	pMM247-pCYC1min-k54-yEGFPgg-CYC1t
pMM322	pMM247-pCYC1min-k55-yEGFPgg-CYC1t
pMM323	pMM247-pCYC1min-k56-yEGFPgg-CYC1t
pMM324	pMM247-pCYC1min-k57-yEGFPgg-CYC1t
pMM325	pMM247-pCYC1min-k58-yEGFPgg-CYC1t

Table S2: List of the plasmids constructed with the Golden Gate method. The acceptor vector pMM247 was assembled with the Gibson method but, for clarity, it is reported here.

Yeast strains used in this work

Strain name	Genotype	5'-UTR ID
byMM2	FY1679-08A	-
byMM15	byMM2 pMM61::URA3	k0
byMM26	byMM2 pMM75::URA3	k1
byMM27	byMM2 pMM76::URA3	k2
byMM56	byMM2 pMM121::URA3	k3
byMM52	byMM2 pMM113::URA3	k4
byMM28	byMM2 pMM77::URA3	k5
byMM29	byMM2 pMM78::URA3	k6
byMM30	byMM2 pMM79::URA3	k7
byMM31	byMM2 pMM83::URA3	k8
byMM32	byMM2 pMM84::URA3	k9
byMM39	byMM2 pMM85::URA3	k10
byMM36	byMM2 pMM86::URA3	k11
byMM115	byMM2 pMM93::URA3	k12
byMM59	byMM2 pMM130::URA3	k13
byMM41	byMM2 pMM97::URA3	k14
byMM42	byMM2 pMM98::URA3	k15
byMM43	byMM2 pMM99::URA3	k16
byMM180	byMM2 pMM100::URA3	k17
byMM53	byMM2 pMM116::URA3	k18
byMM46	byMM2 pMM101::URA3	k19
byMM47	byMM2 pMM104::URA3	k20
byMM48	byMM2 pMM105::URA3	k21
byMM49	byMM2 pMM106::URA3	k22
byMM181	byMM2 pMM107::URA3	k23
byMM51	byMM2 pMM108::URA3	k24
byMM55	byMM2 pMM118::URA3	k25

Table S3: List of yeast strains containing 5'-UTRs from k_0 to k_{25} (plus the original one, byMM2). Plasmids are described in Tables S1.

Strain name	Genotype	5'-UTR ID
byMM64	byMM2 pMM143::URA3	k26
byMM182	byMM2 pMM141::URA3	k27
byMM69	byMM2 pMM150::URA3	k28
byMM70	byMM2 pMM151::URA3	k29
byMM71	byMM2 pMM153::URA3	k30
byMM72	byMM2 pMM154::URA3	k31
byMM114	byMM2 pMM166::URA3	k32
byMM94	byMM2 pMM167::URA3	k33
byMM79	byMM2 pMM175::URA3	k34
byMM74	byMM2 pMM159::URA3	k35
byMM85	byMM2 pMM168::URA3	k36
byMM76	byMM2 pMM158::URA3	k37
byMM80	byMM2 pMM176::URA3	k38

Table S4: List of yeast strains containing 5'-UTRs from k_{26} to k_{38}. Plasmids are described in Tables S1.

Strain name	Genotype	5'-UTR ID
byMM166	byMM2 pMM272::URA3	k39
byMM152	byMM2 pMM273::URA3	k40
byMM153	byMM2 pMM274::URA3	k41
byMM154	byMM2 pMM275::URA3	k42
byMM155	byMM2 pMM276::URA3	k43
byMM156	byMM2 pMM277::URA3	k44
byMM157	byMM2 pMM303::URA3	k45
byMM158	byMM2 pMM304::URA3	k46
byMM159	byMM2 pMM305::URA3	k47
byMM160	byMM2 pMM306::URA3	k48
byMM167	byMM2 pMM307::URA3	k49
byMM168	byMM2 pMM309::URA3	k50
byMM169	byMM2 pMM310::URA3	k51
byMM170	byMM2 pMM311::URA3	k52
byMM171	byMM2 pMM312::URA3	k53
byMM172	byMM2 pMM321::URA3	k54
byMM173	byMM2 pMM322::URA3	k55
byMM174	byMM2 pMM323::URA3	k56
byMM175	byMM2 pMM324::URA3	k57
byMM176	byMM2 pMM325::URA3	k58

Table S5: List of yeast strains containing 5'-UTRs from k_{39} to k_{58}. Plasmids are described in Tables S2.

MFE values

ID	MFE (kcal/mol)
k_0-k_3	**−241.21**
k_4	−241.42
k_5-k_{25}	**−241.21**
k_{26}	−261.39
k_{27}	−247.04
k_{28}	−246.41
k_{29}	−245.97
k_{30}	−244.28
k_{31}	−247.46
k_{32}	**−241.21**
k_{33}	−241.93
k_{34}	−246.59
k_{35}	−242.5
k_{36}-k_{58}	**−241.21**

Table S6: Minimum free energies associated with the mRNA structures of the 59 sequences analyzed in this work.

DNA part sequences

pCYC1min sequence

GCATGCATGTGCTCTGTATGTATATAAAACTCTTGTTTTCTTCTTTTCTCTAAATATTCTTT
CCTTATACATTAGGACCTTTGCAGCATAAATTACTATACTTCTATAGACACACAAACACAA
ATACACACACTAAATTAATA

yEGFP sequence

ATGTCTAAAGGTGAAGAATTATTCACTGGTGTTGTCCCAATTTTGGTTGAATTAGA
TGGTGATGTTAATGGTCACAAATTTTCTGTCTCCGGTGAAGGTGAAGGTGATGCT
ACTTACGGTAAATTGACCTTAAAATTTATTTGTACTACTGGTAAATTGCCAGTTCCA
TGGCCAACCTTAGTCACTACTTTCGGTTATGGTGTTCAATGTTTTGCGAGATACCC
AGATCATATGAAACAACATGACTTTTTCAAGTCTGCCATGCCAGAAGGTTATGTTCA
AGAAAGAACTATTTTTTTCAAAGATGACGGTAACTACAAGACCAGAGCTGAAGTCA
AGTTTGAAGGTGATACCTTAGTTAATAGAATCGAATTAAAAGGTATTGATTTTAAAGA
AGATGGTAACATTTTAGGTCACAAATTGGAATACAACTATAACTCTCACAATGTTTAC
ATCATGGCTGACAAACAAAAGAATGGTATCAAAGTTAACTTCAAAATTAGACACAACA
TTGAAGATGGTTCTGTTCAATTAGCTGACCATTATCAACAAAATACTCCAATTGGTGA
TGGTCCAGTCTTGTTACCAGACAACCATTACTTATCCACTCAATCTGCCTTATCCAAA
GATCCAAACGAAAAGAGAGACCACATGGTCTTGTTAGAATTTGTTACTGCTGCTGGT
ATTACCCATGGTATGGATGAATTGTACAAATAA

yEGFPgg sequence

ATGTCTAAAGGTGAAGAATTATTCACTGGTGTTGTCCCAATTTTGGTTGAATTAGATG
GTGATGTTAATGGTCACAAATTTTCTGTCTCCGGTGAAGGTGAAGGTGATGCTACTTA
CGGTAAATTGACCTTAAAATTTATTTGTACTACTGGTAAATTGCCAGTTCCATGGCCAA
CCTTAGTCACTACTTTCGGTTATGGTGTTCAATGTTTTGCGAGATACCCAGATCATAT
GAAACAACATGACTTTTTCAAGTCTGCCATGCCAGAAGGTTATGTTCAAGAAAGAAC
TATTTTTTTCAAAGATGACGGTAACTACAAGACCAGAGCTGAAGTCAAGTTTGAAGG

TGATACCTTAGTTAATAGAATCGAATTAAAAGGTATTGATTTTAAAGAAGATGGTAACA
TTTTAGGTCACAAATTGGAATACAACTATAACTCTCACAATGTTTACATCATGGCTGAC
AAACAAAAGAATGGTATCAAAGTTAACTTCAAAATTAGACACAACATTGAAGATGGTTC
TGTTCAATTAGCTGACCATTATCAACAAAATACTCCAATTGGTGATGGTCCAGTCTTGT
TACCAGACAACCATTACTTATCCACTCAATCTGCCTTATCCAAAGATCCAAACGAAAAG
AGGGACCACATGGTCTTGTTAGAATTTGTTACTGCTGCTGGTATTACCCATGGTATGGA
TGAATTGTACAAATAA

CYC1t sequence

CATGTAATTAGTTATGTCACGCTTACATTCACGCCCTCCCCCCACATCCGCTCTA
ACCGAAAAGGAAGGAGTTAGACAACCTGAAGTCTAGGTCCCTATTTATTTTTTTA
TAGTTATGTTAGTATTAAGAACGTTATTTATATTTCAAATTTTTCTTTTTTTTCTGTA
CAGACGCGTGTACGCATGTAACATTATACTGAAAACCTTGCTTGAGAAGGTTTTG
GGACGCTCGAAGGCTTTAATTTGCAAGCT

Figures

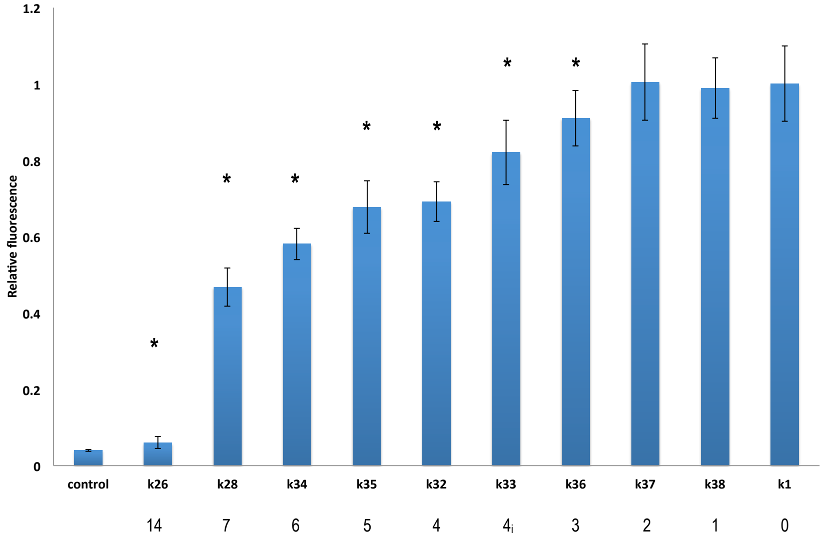

Figure S1: Multiple point mutations to guanine. The ratio between the fluorescence level of ten synthetic 5'-UTRs and the one of k_1 is plotted. Below the leader sequence name the number of guanines present along the 15-nucleotide-long end of our synthetic 5'-UTRs is given. k_{26} contains a cytosine at position -7. As for the other leader sequences, all the guanines are inside the upstream region. 4_i refers to the *intermixed* configuration of guanines and adenines inside k_{33}. Fluorescence increases almost linearly by reducing the number of guanines. Asterisks indicate statistically significant difference with respect to k_1.

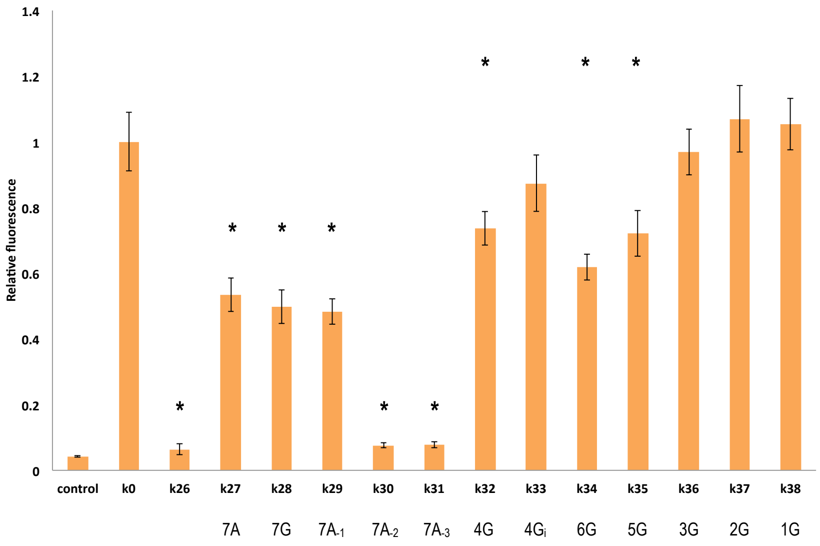

Figure S2: Multiple point mutations to guanine. The ratio between the fluorescence level of the synthetic 5'-UTRs from k_{26} to k_{38} and the one of k_0 is plotted. Below the leader sequence name (from k_{27} to k_{38}) the number of adenines or guanines present in the upstream region is given. The subscripts -1, -2, and -3 indicate that an adenine is present in the extended Kozak sequence only at the corresponding position. The subscript i refers to the *intermixed* configuration of guanines and adenines inside k_{33}. Asterisks point out statistically significant difference with respect to k_0. k_{33} and k_{36} show statistically significant difference with respect to k_1 (see the main text) but not to k_0. This is due to the fact that the fluorescence level of k_0 is only, approximatively, 94 % of the one of k_1.

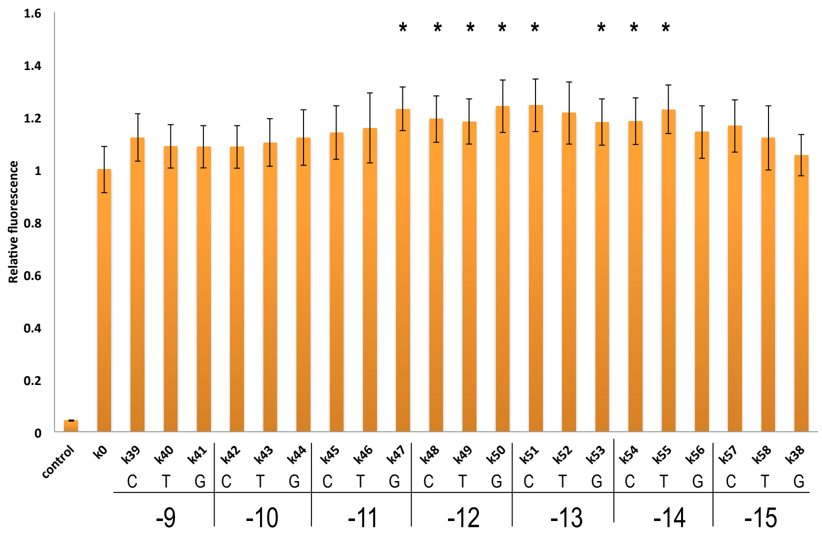

Figure S3: Effect of point mutations, along the upstream region, on fluorescence expression. Fluorescence levels relative to the one of k_0 are presented. Below the name of each synthetic leader sequence the nucleotide that replaced an adenine in k_1 and the position at which the mutation took place are given. Asterisks denote statistically significant difference with respect to k_0.

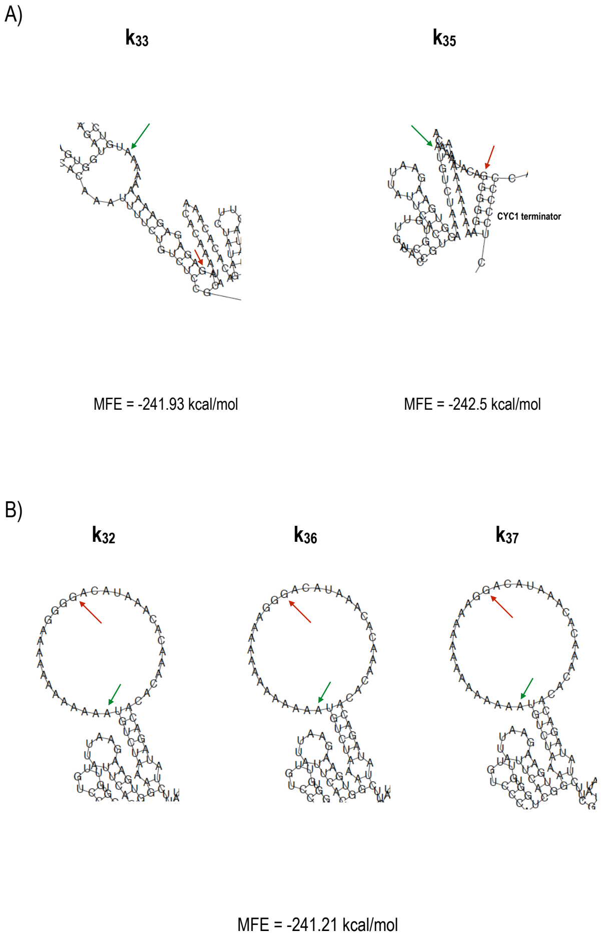

Figure S4: mRNA secondary structures. A) The *intermixed* configuration of k_{33}, where four guanines alternate with adenines in the upstream region, give rise to a stem that involves 10 nucleotides between position -15 and -5. However, this mRNA secondary structure has a higher MFE with respect to k_{35}, where only 6 nucleotides of the upstream region are involved into a stem. k_{33} let also register a fluorescence level higher than the one of k_{35}. B) Despite the presence of multiple guanines along the upstream region, the mRNA secondary structures of k_{32}, k_{36}, and k_{37} show the giant hairpin, as they are computed by RNAfold.
